# Current Hospital Topics

**Published:** 1908-03-21

**Authors:** 


### HOSPITAL ADMINISTRATION.

### CONSTRUCTION AND ECONOMICS.

### CURRENT HOSPITAL TOPICS.

**The late Mr. H. L. Bischoffsheim.**

Mr. H. L. Bischoffsheim's death will be [illegible]gretted by all who had an intimate knowledge of [illegible]s methods in charity. He belonged to a type [illegible]hich is rare in these days, because most of his [illegible]fts were so made as to keep his name out of the [illegible]apers. For many years of his active life Mr. [illegible]ischoffsheim was in the habit of sending some[illegible]ing to almost every charity which wrote to him for [illegible]upport. In later years, when he came to take an [illegible]tive personal interest in the management of chari[illegible]es, he soon realised that much money might be [illegible]asted by following his old system, and he therefore [illegible]hanged it in favour of one of giving considerable [illegible]ums to approved objects after he had fully investi[illegible]ated them and assured himself of their economic [illegible]nd general excellence. It was in this way that Mr. [illegible]ischoffsheim, after witnessing the unnecessary [illegible]uffering of a man who was injured in a street acci[illegible]ent in the City, became interested in ambulance [illegible]ork. A paper had been read on the subject of [illegible]treet ambulances in London before the Hospitals [illegible]ssociation by Mr. Thomas Ryan, and when Mr. [illegible]ischoffsheim made inquiries of Sir Henry Burdett [illegible]e was handed Mr. Ryan's paper, and it was sug[illegible]ested that he should co-operate by finding the [illegible]oney to work out a practical scheme, which he [illegible]ltimately did. The amount of money which Mr. [illegible]ischoffsheim has contributed to the street ambu[illegible]ance system in London is very considerable, and [illegible]is generosity to it, and the personal service which [illegible]e rendered by undertaking to supervise the whole [illegible]f the arrangements and the management, in [illegible]onjunction with a small committee of which Mr. Ryan is the honorary secretary, is beyond all praise. Mr. Bischoffsheim also founded, [illegible]rected, and paid for a sanatorium for consump[illegible]ives, which has been one of the most successful of [illegible]ts kind. In this work he had the warm sympathy and co-operation of Mrs. Bischoffsheim, who has [illegible]isplayed genuine interest in this branch of institu[illegible]ional work. We had the pleasure of knowing Mr. Bischoffsheim intimately for many years, and can say from personal knowledge that there were few men in the City who had a kinder heart or who were ever more ready to lend a helping hand to every deserving case where a little monetary help could be proved to offer the prospect of permanent benefit. It is one of the drawbacks of present-day life in the City of London that the Limited Liability Acts have gradually extinguished private firms, and so benefactors of the type of character and usefulness of the late Mr. H. L. Bischoffsheim tend to grow fewer and fewer each year, to the infinite loss of the community and of all that is best in City life.

**The General Hospital, Birmingham.**

This hospital is one of the best of provincial institutions. It has always occupied a deservedly high place for the efficiency of its management, and the 128th annual report just issued proves that the present managers are worthily maintaining the traditions of their predecessors. The out-patient hall is one of the best ventilated and most comfortable in the hospital world. The plenum system lends itself to the ventilation of large halls in a crowded condition, but it is ill suited for resident quarters in a big hospital, and should never, in our opinion, be applied to children's wards. We have always felt that the plenum system is not applicable to hospital buildings in a temperate climate like that of the British Islands, and we consider the working of the General Hospital on the whole abundantly justifies this view. The report records that the work in the out-patient and casualty departments has continued to engage the serious attention of the committee. A special committee appointed for the purpose has been at work upon this knotty point in hospital relief for a considerable time, and the committee, on the recommendation of the medical staff, have decided to instruct their medical officers to refer elsewhere trivial cases, after giving first aid, and to deal with chronic cases which do not require hospital treatment by handing them on to general practitioners, clubs, dispensaries, or the Poor-law. This new departure will be watched with interest by many other hospitals, and we hope that the new plan may prove in practice a complete success. Economically this hospital is well administered. The average cost of each in-patient is three shillings less than it was five years ago. The average weekly cost of each resident member of the staff also snows a reduction, whilst the cost per bed occupied per annum on the total ordinary expenditure was, in round figures, £79 in 1907, as compared with £83 in 1903. Another interesting feature is that the number of days each in-patient was resident has fallen from 22½ to 20 within the quinquennium. The Birmingham public do not seem, however, to appreciate adequately these economical features in the management. Thus the annual subscriptions, which amounted to upwards of £8,000 in 1903, have fallen to £7,508 in 1907. A drop of one-sixteenth in this important source of revenue is a serious matter, and we are glad to see that the committee calls attention to it in the annual report. Wake up men of Birmingham, and exhibit more intelligence by providing more cash in annual subscriptions!

